# Choir versus Solo Singing: Effects on Mood, and Salivary Oxytocin and Cortisol Concentrations

**DOI:** 10.3389/fnhum.2017.00430

**Published:** 2017-09-14

**Authors:** T. Moritz Schladt, Gregory C. Nordmann, Roman Emilius, Brigitte M. Kudielka, Trynke R. de Jong, Inga D. Neumann

**Affiliations:** ^1^Department of Behavioural and Molecular Neurobiology, University of Regensburg Regensburg, Germany; ^2^University Choir Regensburg, University of Regensburg Regensburg, Germany; ^3^Department of Medical Psychology, Psychological Diagnostics and Research Methodology, University of Regensburg Regensburg, Germany

**Keywords:** choir singing, mood, oxytocin, cortisol, saliva

## Abstract

The quantification of salivary oxytocin (OXT) concentrations emerges as a helpful tool to assess peripheral OXT secretion at baseline and after various challenges in healthy and clinical populations. Both positive social interactions and stress are known to induce OXT secretion, but the relative influence of either of these triggers is not well delineated. Choir singing is an activity known to improve mood and to induce feelings of social closeness, and may therefore be used to investigate the effects of positive social experiences on OXT system activity. We quantified mood and salivary OXT and cortisol (CORT) concentrations before, during, and after both choir and solo singing performed in a randomized order in the same participants (repeated measures). Happiness was increased, and worry and sadness as well as salivary CORT concentrations were reduced, after both choir and solo singing. Surprisingly, salivary OXT concentrations were significantly reduced after choir singing, but did not change in response to solo singing. Salivary OXT concentrations showed high intra-individual stability, whereas salivary CORT concentrations fluctuated between days within participants. The present data indicate that the social experience of choir singing does not induce peripheral OXT secretion, as indicated by unchanged salivary OXT levels. Rather, the reduction of stress/arousal experienced during choir singing may lead to an inhibition of peripheral OXT secretion. These data are important for the interpretation of future reports on salivary OXT concentrations, and emphasize the need to strictly control for stress/arousal when designing similar experiments.

## Introduction

The neuropeptide oxytocin (OXT) has been found to promote social affiliation and bond formation, to reduce anxiety, and to dampen the stress response in many mammalian species, including humans (Donaldson and Young, 2008; Lee et al., 2009; Neumann and Landgraf, 2012; Young, 2015). Moreover, OXT is known to mediate the stress-buffering effects of close social interactions (Smith and Wang, 2014), and the anxiolytic effects of sexual interactions (Waldherr and Neumann, 2007). A well-functioning OXT system is, therefore, of great importance for day-to-day life, which is emphasized by the association of OXT system imbalances with socio-emotional dysfunctions in patients with autism spectrum disorder, borderline personality disorder, and anxiety disorders, especially social anxiety disorder (for reviews see Meyer-Lindenberg et al., 2011; Neumann and Landgraf, 2012; Cochran et al., 2013; Neumann and Slattery, 2016).

Quantifying OXT concentrations in central and peripheral body fluids has become a popular tool with the aim to assess the general activity of the OXT system in both healthy and clinical populations, either at rest or after an experimental challenge (Crockford et al., 2014; de Jong et al., 2015; Rutigliano et al., 2016). OXT concentrations in cerebrospinal fluid (CSF) samples optimally reflect changes in central OXT neurotransmission responsible for socio-emotional functions (Jokinen et al., 2012; Kagerbauer et al., 2013; Carson et al., 2015). However, CSF sampling requires invasive lumbar puncture and trained medical personnel, which makes this method relatively expensive and logistically difficult (e.g., recruitment of test subjects and limited number of samples). Most studies have therefore relied on the quantification of OXT concentrations in peripheral fluids such as blood and, more recently, saliva, which are much easier to obtain (especially saliva). Despite the fact that many physiological stimuli, such as birth, suckling during lactation, sexual stimulation, and stress have been shown to activate both peripheral secretion of OXT into blood as well as release within distinct brain regions as studied mainly in laboratory rodents, one has to keep in mind that the secretion of OXT from the pituitary may also occur functionally and temporarily independent from the release of OXT within the brain (Landgraf and Neumann, 2004; Neumann and Landgraf, 2012; Torner et al., 2017). In other words, changes in OXT concentrations in blood plasma and saliva may occur without any concomitant changes in central OXT neurotransmission, and vice versa. In this context it is of interest to note that a recent meta-analysis that included studies conducted in humans, non-human primates, sheep, rats, and mice confirmed that OXT concentrations in central fluids (CSF or extracellular fluid) correlated positively with OXT concentrations in blood plasma after experimentally induced stress, though not at baseline (Valstad et al., 2017).

Quantifying OXT concentrations in saliva is currently gaining popularity due to the user-friendly sampling method (Carter et al., 2007; de Jong et al., 2015), following in the footsteps of the well-validated analysis of salivary cortisol (CORT; Kirschbaum and Hellhammer, 1994). The precise relationship between OXT concentrations in CSF, blood, and saliva remains to be elucidated, but it has been shown that situations known to induce OXT release in the brain and blood, i.e., running, masturbation and social stress, also cause rapid and sharp increases in salivary OXT concentrations (de Jong et al., 2015).

Thus far, peripheral OXT secretion has been reported in response to intense social experiences including parent-child interactions, recalling a romantic event, warm contact with a loved one, and gossiping with close friends (Grewen et al., 2005; Gonzaga et al., 2006; Feldman et al., 2010; Kim et al., 2014; Krause et al., 2016; Lebowitz et al., 2016; Brondino et al., 2017). However, OXT is also secreted in response to various kinds of stress (Pierrehumbert et al., 2010; de Jong et al., 2015; Brown et al., 2016). Therefore, if stress is not properly controlled for, an increase in peripheral OXT concentrations may be falsely interpreted as a “pro-social” rather than a stress response.

The current experiment was designed to assess salivary OXT and CORT concentrations in male and female healthy volunteers in response to a positive social challenge: choir singing. In general, choir singing is known to improve mental and physical health and overall quality of life (Clift et al., 2010; Chanda and Levitin, 2013; Coulton et al., 2015). A single choir singing session improves mood and increases trust, cooperative behavior, and feelings of social closeness in the singers (Anshel and Kipper, 1988; Weinstein et al., 2016) while reducing anxiety and other negative feelings (Kreutz et al., 2004; Kreutz, 2014; Fancourt et al., 2016). OXT has been proposed to represent the biological link between the intense social experience of choir singing and its positive psychosocial effects (Chanda and Levitin, 2013; Kreutz, 2014). However, previous studies reported inconsistent results, i.e., increased or decreased salivary OXT concentrations (Kreutz, 2014; Fancourt et al., 2016) and stable plasma OXT levels (Keeler et al., 2015) in response to choir singing. Similarly, some studies found that choir singing resulted in reduced activity of the hypothalamic-pituitary-adrenal (HPA) axis as reflected by lower levels of adrenocorticotropic hormone (ACTH) and CORT in plasma (Beck et al., 2000; Keeler et al., 2015) and saliva (Fancourt et al., 2016), whereas others could not confirm this in saliva samples (Kreutz et al., 2004; Kreutz, 2014). Interestingly, one study reported modest increases in plasma OXT and CORT concentrations in amateur and professional singers during an *individual* singing lesson with a teacher. In addition, both groups showed improved mood after the lesson: they felt more joyful, energetic and relaxed as measured by a Visual Analog Scale (Grape et al., 2002).

So far, no study has directly compared peripheral OXT and CORT secretion between choir and solo singers. We designed an experiment that was aimed to induce as little stress/arousal as possible and that allowed us to compare the neuroendocrine effects of a 20-min choir singing session (social stimulus) versus a 20 min solo singing session (non-social stimulus) within the same subjects (repeated measures). We predicted that both choir and solo singing would improve mood as quantified by the *State and Trait Anxiety and Depression Inventory* (STADI; Laux et al., 2013), with choir singing exerting the strongest positive effect. We also predicted that the social experience of choir singing would result in markedly increased OXT concentrations compared with both basal levels and with solo singing, whereas the secretion of CORT would be higher under solo versus choir singing conditions.

## Materials and Methods

### Participants

A total of 38 student chorists were recruited from the University Choir of the University of Regensburg. All singers had been part of the choir for at least 5 months with weekly rehearsals and were familiar with the music literature used in this study. None of the study participants currently experienced a period of stress, such as ongoing exams or relational problems. Each participant underwent two testing sessions at two different days, between 18:00 h and 20:30 h. The complete procedure was performed in two cohorts: one with 21 participants (males: *n* = 9, females: *n* = 12, median years of age: 22, range: 19–26) and one with 17 participants (males: *n* = 8, females: *n* = 9, median years of age: 23, range: 18–29). For the first cohort of singers, the order of both experimental sessions was randomly assigned; 50% of the participants underwent the solo singing session 4 days before, 50% 2 days after the choir session. As we found no effect of order in the first cohort, in the second cohort all singers first performed the choir session followed by the solo singing session 4 days later. All participants received detailed verbal and written information on the study procedure and gave their written consent to their participation. Due to the non-invasive and voluntary procedures, the study was exempted from evaluation by the ethical committee of the University of Regensburg.

### Experimental Procedures

All test sessions took place at the University of Regensburg. Participants were asked to refrain from consuming food and drinks, brushing teeth, or physical activity from at least 1 h before the start of the experimental procedure. For the choir condition, all participants were seated together in their usual rehearsal room throughout the experiment and were instructed to refrain from social interaction other than singing. The 20-min singing task was conducted by RE, the regular conductor of the choir, in order to preserve a naturalistic character of the choir experience. It was furthermore emphasized that this was a normal rehearsal and the performance of the participants was not evaluated in any way. Experimenters monitored the timely distribution and collection of pre-coded Salivettes (Sarstedt, Nümbrecht, Germany) and the relaxed experimental ambiance. For the solo condition, each participant was seated alone in a small room and provided with written instructions for singing and saliva sampling procedures, a timer, a coolbox with ice and five pre-coded Salivettes. Sampling was completed without intervention from the experimenters.

In both choir and solo sessions, the singing task included identical excerpts of the oratorio *Messiah* by G. F. Händel (HWV 56; cohort 1), or of parts IV, V and VI of the *Christmas Oratorio* by J. S. Bach (BWV 248; cohort 2), which were familiar to the participants. A mixed, 20 min program containing both homophonous and polyphonic four-part chorales was selected from those pieces (see Supplementary Material). Participants were instructed to perform the singing task identically during both solo and choir conditions.

### Saliva Sampling

For each saliva sample, the participants were instructed to gently chew on the cylindrical swab of a Salivette for approximately 1 min. The swab was returned to the Salivette tube and placed on ice. After collection of the last (Post) sample, all Salivettes were immediately transported to a laboratory freezer and stored at −20°C until quantification procedures.

**Figure 1:**
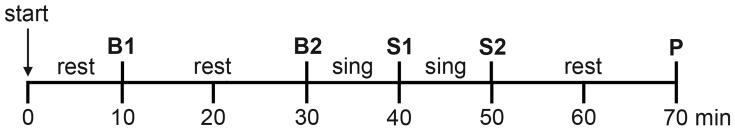
Experimental timeline for the collection of saliva samples. Basal samples (B1 and B2) were collected after 10 and 30 min of resting. Singing samples (S1 and S2) were collected after 10 and 20 min of singing. One post-singing sample (P) was collected 20 min after cessation of singing. The timeline was identical for the solo and choir conditions.

The time plan for saliva sampling, which was identical under choir and solo conditions, is shown in Figure 1. Basal samples were collected 10 min (Basal 1) and 30 min (Basal 2) after beginning of the experiment while participants were quietly resting. Immediately after the collection of the Basal 2 sample, the participants started singing, and saliva samples were collected after 10 min of continuous singing (Singing 1) and again after 10 additional min of continuous singing (Singing 2). Participants did not resume singing after collection of the Singing 2 samples; 20 min later a post-singing sample was collected (Post). Participants remained seated throughout the procedure to avoid physical strain.

### Questionnaires

Prior to each session, participants received envelopes containing personal questionnaires and two sets of the STADI. Personal questionnaires on basic demographics (sex, age), singing background, affiliation to the choir, physical and mental health status, smoking habits, medication, use of hormonal contraceptives, stress and preceding physical activity and food consumption were administered in order to assess subjective singing expertise and social engagement in the choir as well as influencing factors for the hormonal measurements. In addition, the STADI was used to assess trait anxiety and depression, as well as emotional states prior and after each singing task (Renner et al., 2016). Participants completed the questionnaires during the period of rest prior to and after choir and solo singing, respectively. Since none of the participants deviated from the norm for trait anxiety and depression, only the results for STADI state (change from prior to after singing) are discussed here. This inventory consists of 20 statements, with which the participants have to agree or disagree on a 4-point Likert-type rating scale. The statements assess *excitement* (the affective component of anxiety, for example: “my heart is beating fast”), *worry* (the cognitive component of anxiety, for example: “I am brooding over my situation”), *dysthymia* (negative affect as a marker of depression, for example: “I am in a bad mood”) and *euthymia* (positive affect as a reversed marker of depression, for example: “I feel good”).

### Quantification of Salivary OXT and CORT

Salivettes were thawed and centrifuged at 3000 rpm for 5 min, which resulted in a clear supernatant of low viscosity. Saliva samples from the first cohort were used for quantification of OXT concentrations alone, whereas saliva samples from the second cohort were split in two to enable a combination of OXT and CORT measurements. Quantification of OXT concentrations was commercially performed by radioimmunoassay (RIAgnosis, Munich, Germany) with a sensitivity of 0.1 to 0.5 pg/sample as described previously (de Jong et al., 2015). Intra- and inter-assay coefficients for OXT were <10% and <12%, respectively. Quantification of CORT concentrations was performed using a commercially available chemiluminescence immunoassay with high sensitivity (IBL International, Hamburg, DE via Dresden Lab Service, Dresden, Germany). Intra- and inter-assay coefficients for cortisol were below 8%.

### Statistics

All data were analyzed using SPSS 21.0 (IBM Corp., Armonk, NY, USA) with *p* ≤ 0.05 considered statistically significant. All data are shown as means ± SEM.

STADI-state results for excitation, worry, happiness (euthymy), and sadness (dystymy) were analyzed using a two-way analysis of variance (ANOVA) for repeated measures with time (before and after singing) and context (solo and choir) as within-subjects factors. A three-way ANOVA with sex as a between-subjects factor was also performed. *Post hoc* pair-wise comparisons were made using paired *t*-tests.

OXT samples were collected from two different cohorts. We therefore compared OXT samples collected during choir singing between the two cohorts by performing a two-way ANOVA; the interaction effect between time (before and after signing) and cohort (Händel and Bach) did not reach statistical significance (*p* = 0.78), wherefore we pooled both sets of results. Since salivary OXT and CORT concentrations were not normally distributed and basal concentrations showed considerable individual variability, these values were normalized to percentage of their corresponding baseline as follows: (value x)/((value of Basal 1 + value of Basal 2)/2) * 100%. Relative changes in salivary OXT and CORT were then analyzed using a two-way ANOVA for repeated measures, using time (the five consecutive samples) and context (choir and solo) as within-subjects factors. A three-way ANOVA with sex as a between-subjects factor was also performed. Huyn-Feldt corrections were made if Mauchly’s test indicated that the assumption of sphericity was violated. Significant main and interaction effects were followed up with Bonferroni-corrected *post hoc* pair-wise comparisons.

Pearson’s correlation coefficients were analyzed for absolute OXT and CORT concentrations in Basal 2 and Singing 2 samples collected before and after 20 min of choir and solo singing.

## Results

### STADI-State

**Figure 2:**
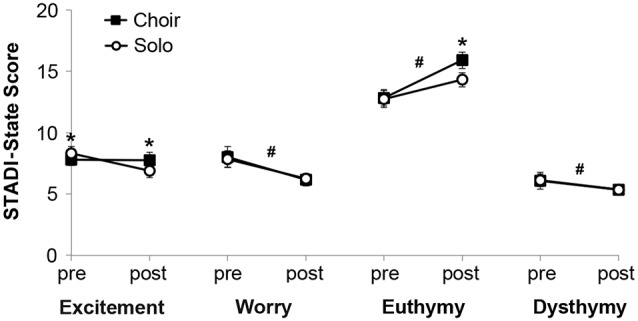
Mood states measured with the State and Trait Anxiety and Depression Inventory (STADI)-state before (“pre”) and after (“post”) 20 min of choir or solo singing, respectively. *p < 0.05 between corresponding choir and solo values. ^#^*p* < 0.05 between corresponding pre and post values.

Analysis of STADI-state outcomes (Figure 2) revealed main effects of time on the parameters worry (decreased after singing, *F*_(1)_ = 47.28, *p* < 0.001), happiness (increased after singing, *F*_(1)_ = 100.96, *p* < 0.001) and sadness (decreased after singing, *F*_(1)_ = 10.16, *p* = 0.003), with a trend for excitement (decreased after singing, *F*_(1)_ = 4.04, *p* = 0.052). There were no main effects of context, but there were interaction effects of time and context on the parameters excitement (*F*_(1)_ = 6.51, *p* = 0.015) and happiness (*F*_(1)_ = 5.27, *p* = 0.028). *Post hoc* pair-wise comparisons indicated that participants were more excited prior to solo singing compared with choir singing, but this excitement dropped after solo singing below the stable choir singing values (*p* = 0.014). Participants did not differ in happiness prior to solo versus choir singing, but they were happier after choir compared with solo singing (*p* = 0.007) None of these effects were significantly influenced by sex.

### Salivary OXT Levels

**Figure 3:**
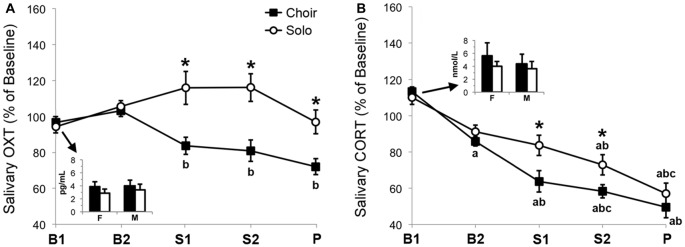
Relative change in **(A)** salivary oxytocin (OXT) concentrations (*n* = 38) and **(B)** salivary cortisol (CORT) concentrations (*n* = 17) in two basal samples (B1, B2), two singing samples (S1, S2) and one post-singing sample (P) collected before, during and after 20 min of choir or solo singing, calculated as percentage of baseline (= mean of B1 + B2 values). Inserts depict absolute concentrations of OXT and CORT in B1 samples of female (F) and male (M) participants. *p < 0.05 between corresponding choir and solo values. ^a/b/c^*p* < 0.05 versus corresponding ^a^B1, ^b^B2, or ^c^S1 values.

Salivary OXT levels (Figure 3A) changed significantly over time (*F*_(3.10)_ = 3.66, $\eta_{\text{p}}^{\text{2}}$ = 0.090, *p* = 0.007), and a main effect of context emerged (*F*_(1)_ = 1441.6, $\eta_{\text{p}}^{\text{2}}$ = 0.353, *p* < 0.001). Moreover, there was a significant interaction between time and context (*F*_(4)_ = 7.27, $\eta_{\text{p}}^{\text{2}}$ = 0.164, *p* < 0.001).

*Post hoc* pair-wise comparisons revealed that in the context of choir singing, salivary OXT concentrations decreased over time and were lower at Singing 1 (*p* = 0.019) and Singing 2 (*p* = 0.041) compared with Basal 2. OXT concentrations were lower in the Post sample compared to both Basal 1 and Basal 2 (*p* < 0.001).

During and after solo singing, salivary OXT concentrations showed a mild, non-significant increase relative to basal. Consistently, pair-wise comparisons between the two contexts revealed that salivary OXT levels were lower during choir singing than during solo singing in samples Singing 1 (*p* = 0.002), Singing 2 (*p* < 0.001) and Post (*p* = 0.002). None of these effects were influenced by sex.

### Salivary CORT Levels

Salivary CORT levels (Figure 3B) changed significantly over time (*F*_(2.95)_ = 52.95, $\eta_{\text{p}}^{\text{2}}$ = 0.768, *p* < 0.001) and a main effect of context emerged (*F*_(1)_ = 5.80, $\eta_{\text{p}}^{\text{2}}$ = 0.266, *p* = 0.028), whereas the interaction effect between time and context did not reach statistical significance (*F*_(3.31)_ = 2.22, $\eta_{\text{p}}^{\text{2}}$ = 0.122, *p* = 0.091).

*Post hoc* pair-wise comparisons showed that in the context of choir singing, salivary CORT concentrations decreased continuously over time and were lower at Basal 2 compared with Basal 1 (*p* = 0.001), at Singing 1 compared with both Basal 1 (*p* < 0.001) and Basal 2 (*p* = 0.001), at Singing 2 compared with Basal 1 and 2 (*p* < 0.001) and with Singing 1 (*p* = 0.036), and at Post compared with Basal 1 and 2 (*p* < 0.001).

The drop in salivary CORT concentrations was more gradual in the context of solo singing with lower levels at Singing 2 compared with Basal 1 (*p* = 0.004) and Basal 2 (*p* = 0.011), and at Post compared with Basal 1, Basal 2 and Singing 1 (*p* ≤ 0.001). Pair-wise comparisons between the two conditions revealed that salivary CORT levels were lower during choir singing than during solo singing at Singing 1 (*p* = 0.025) and Singing 2 (*p* = 0.010).

The change in salivary CORT concentrations over time was influenced by sex, independent of condition as shown by a three-way ANOVA (time × sex: *F*_(4)_ = 9.38, $\eta_{\text{p}}^{\text{2}}$ = 0.385, *p* < 0.001). Descriptively, salivary CORT decreased more steeply in men compared to women in both choir-singing and solo-singing contexts (data not shown).

### Correlations

**Figure 4:**
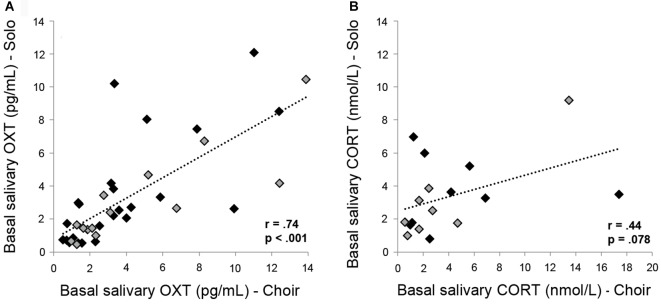
Correlations between** (A)** basal OXT concentrations and **(B)** basal CORT concentrations measured in saliva sampled from the same subjects at two different days, with 2–5 days separating the two samples. Black markers represent female subjects; gray markers represent male subjects.

Pearson’s correlation coefficients were analyzed for absolute OXT and CORT concentrations in basal samples (Basal 2) and in samples collected after 20 min of choir and solo singing (Singing 2). OXT and CORT concentrations measured in the same saliva samples did not correlate significantly with one another in the context of choir singing (Basal 2 samples: *r* = 0.286, *p* = 0.266; Singing 2 samples: *r* = 0.131, *p* = 0.615) or solo singing (Basal 2 samples: *r* = −0.241, *p* = 0.351; Singing 2 samples: *r* = −0.171, *p* = 0.527), indicating independent secretion both at baseline and in response to singing. Individual salivary OXT concentrations taken at two different experimental days correlated highly and significantly both under basal conditions (Basal 2 samples; *r* = 0.737, *p* < 0.001; Figure 4A) as well as in response to choir/solo singing (Singing 2 samples; *r* = 0.905, *p* < 0.001, data not shown). Individual salivary CORT concentrations only showed a trend towards a positive correlation prior to solo versus choir singing under basal conditions (Basal 2 samples; *r* = 0.439, *p* = 0.078; Figure 4B; nota bene: removing the two outliers reduced the positive correlation coefficient to *r* = 0.221 (*p* = 0.43)). This trend disappeared after singing (Singing 2 samples; *r* = 0.367, *p* = 0.162, data not shown).

## Discussion

The present experiment was designed to compare the psychological (STADI-state) and neuroendocrine (salivary OXT and CORT concentrations) effects of 20 min of choir singing versus solo singing in order to dissect the pro-social component from the stress-reducing component of singing.

The results show that 20 min of either choir or solo singing is sufficient to increase happiness and decrease sadness and worry, which is consistent with previous findings using visual analog scales (Grape et al., 2002; Fancourt et al., 2016) or questionnaires (Kreutz et al., 2004; Kreutz, 2014; Weinstein et al., 2016) to assess mood. The present data show, for the first time, that the positive effects of singing are more pronounced after choir singing compared with solo singing in the same participants. In addition, whereas feelings of excitement remained stable during choir singing, participants reported high excitement prior to solo singing, which then dropped significantly after solo singing.

Choir and solo singing also had differential effects on salivary OXT concentrations. Whereas salivary OXT levels mildly increased after solo singing (116% of basal), they were significantly reduced after choir singing (81% of basal), independent of sex. The results for the solo condition are in line with a previous finding (Grape et al., 2002), which showed a mild, but significant 125% increase in plasma OXT concentrations in both amateur and professional singers (but note that a currently criticized quantification method was used; see McCullough et al., 2013). In addition, our results for choir singing are in agreement with a recent study reporting a reduction in salivary OXT levels (75% of basal) after 70 min of choir rehearsal, including learning new, unidentified songs (Fancourt et al., 2016). Combined, our results do not support the hypothesis that the social experience of choir singing promotes the secretion of OXT from the neurohypophysis into circulation and eventually in saliva. This hypothesis was based on two lines of reasoning: (i) an earlier study reporting an increase in salivary OXT concentrations (139% of basal) after 30 min of choir rehearsal, including 10 min of warm-up exercises and repeatedly singing the pop-song “California Dreaming” (Kreutz, 2014); and (ii) reports of other positive social experiences resulting in OXT secretion, such as intense parent-child interactions, massage, gossiping with friends, and loving interactions between participants and their dogs (Grewen et al., 2005; Gonzaga et al., 2006; Feldman et al., 2010; Beetz et al., 2013; Kim et al., 2014; Tsuji et al., 2015; Krause et al., 2016; Lebowitz et al., 2016; Brondino et al., 2017). Of course, it may be that positive bilateral interactions or the powerful intimate bond between parents and their children, romantic partners, or dogs and their owners are needed to trigger the OXT system to a quantifiable extent—aspects that are missing from choir singing.

It is possible that increases or decreases in stress/arousal, rather than social experience, drives salivary OXT concentrations. In other words, singing may cause an increase (or a decrease) in stress signals from the brain stem and/or amygdala to the hypothalamus, resulting in the activation (or quiescence) of the HPA axis and the OXT system. If this is true, it can be expected that CORT responses resemble OXT responses to singing. This was indeed the case in previous studies: Kreutz (2014) reported an increase in both OXT and CORT concentrations after choir singing (but note that the reported CORT values were supraphysiologically high), whereas Fancourt et al. (2016) reported a decrease in both OXT and CORT concentrations. In the present study, choir singing also coincided with a marked reduction in both OXT and CORT concentrations, whereas solo singing mildly triggered both the HPA axis and the OXT system relative to choir singing. It needs to be noted, however, that in the absence of a non-singing control group it cannot be excluded that the CORT curve represents the tail end of the circadian peak just before it reaches the nadir at around 16 h after awakening (Miller et al., 2016).

Interestingly, participants reported a reduction in state excitement in response to solo, but not choir singing, despite having higher salivary CORT levels. Of course, excitement is not synonymous with stress/HPA axis activity and the lower scores may have been based on STADI state items not correlated with CORT secretion. Furthermore, there is a known time-lag between psychological and endocrine responses to different tasks, potentially leading to reduced covariance (Schlotz et al., 2008). It is also possible that participants experienced an increase in excitement during solo singing followed by a sudden drop at the end of the task (for example due to relief), rather than a gradual drop throughout the procedures.

The decrease in CORT levels was overall more pronounced in male compared with female participants. This is in contrast to Grape et al. (2002), who reported higher plasma CORT concentrations in men compared to women in response to solo singing, and to other studies showing a more pronounced CORT response to social stress in men (Kirschbaum et al., 1999). In addition, the previously demonstrated influence of hormonal contraceptives and menstrual cycling on salivary CORT and, to a lesser extent, OXT secretion in women (Kirschbaum et al., 1999; de Jong et al., 2015) was not seen in the present study. This may be due to the small sample size.

Although salivary OXT and CORT concentrations on average followed a similar curve, individual values measured in the same samples did not significantly correlate with one another. It is of note that the chemical nature of both hormones is likely to influence the speed by which they will transfer from blood to saliva; the lipophilic layers of the capillaries are more permeable for steroid hormones (such as CORT) than for hydrophilic peptides (like OXT; Gröschl, 2008). However, even OXT (which is therefore likely to diffuse slower than CORT) is able to reach the saliva within 10 min, as shown for other challenges such as running, sexual self-stimulation, and the Trier Social Stress Test (de Jong et al., 2015), suggesting that under the present conditions, there was no direct causal relationship between these markers (e.g., the HPA axis triggering the OXT system or vice versa). A recent meta-analysis concluded that basal plasma concentrations of OXT and CORT tend to correlate positively, but only if participants anticipate a novel or stressful experience, i.e., when participants are considerably stressed at the time of measurement (Brown et al., 2016). Since we clearly aimed for a stress-free procedure in the present experiment, especially with respect to sample collection, the lack of a significant correlation is no surprise.

Interestingly, basal and post-singing absolute OXT concentrations were remarkably stable within subjects. Thus, OXT concentrations prior to and after choir singing correlated highly and positively with OXT concentrations prior to and after solo singing (sampled in random order). These findings suggest that salivary OXT concentrations, though quite variable between subjects, do not fluctuate significantly from 1 day to the next within subjects. It also indicates that salivary OXT is a reliable marker for the individual OXT system, as has been shown before for plasma OXT (Bendix et al., 2015). Within-subject correlations were weaker in CORT concentrations, with only a trend toward a positive relationship between basal values, suggesting a higher level of day-to-day fluctuations.

The present study has several limitations. The inclusion of a control condition, such as 20 min of quietly sitting would profile the temporal dynamics of CORT and OXT secretion under resting conditions, and could indicate, whether singing actively inhibits OXT and CORT secretion. Furthermore, another objective measure of arousal, such as heart rate or blood pressure, would have been useful to test the hypothesis that reductions in OXT and CORT concentrations are both a function of significant relaxation during singing. A rather methodological issue concerns the fact that singing may have altered the amount of saliva readily available in the mouth, which could have influenced OXT and CORT concentrations. Therefore, our findings need to be confirmed in plasma samples. Finally, as for all studies assessing peripheral OXT concentrations, it is important to emphasize that these responses may not correlate with central OXT release in regions relevant for social behaviors, emotionality, and stress regulation (Neumann and Landgraf, 2012; Neumann and Slattery, 2016). In other words, the improved mood and feelings of social closeness reported after choir singing may have been caused by subtle release of OXT in distinct brain regions in the absence of measurable increases in peripheral OXT secretion.

Taken together, the present results confirm our hypothesis that 20 min of either choir or solo singing improve mood, with choir singing exerting the stronger effects, and does not activate the HPA axis. More surprisingly, choir singing resulted in a marked reduction in salivary OXT concentrations, whereas solo singing modestly increased salivary OXT concentrations. The data presented here challenge the view that during choir singing, elevated levels of peripheral OXT mark the induction of a “sociobiological bonding response that is similar to those elicited during intimate social relationships” (Kreutz, 2014). Rather, our results indicate that salivary OXT levels are reduced due to the reduction in stress/arousal experienced during choir singing. The mild increase in OXT concentrations in response to solo singing may have been caused by mild stress, at least in female participants. Our data underline the importance to be careful with: (i) assuming that all social experiences cause a measurable increase in peripheral OXT; and (ii) ignoring the importance to control for HPA axis activity when quantifying peripheral OXT concentrations under basal or stimulated conditions.

## Author Contributions

TMS, GCN and IDN designed the experiments; TMS, GCN, RE and TRJ acquired the data; TRJ and BMK analyzed the data, and all authors contributed to data interpretation. TMS, GCN and TRJ drafted the work; BMK and IDN critically revised it for important intellectual content. All authors approved the final version of the manuscript.

## Conflict of Interest Statement

The authors declare that the research was conducted in the absence of any commercial or financial relationships that could be construed as a potential conflict of interest.
