# Supplementary material for: Choir versus Solo Singing: Effects on Mood, and Salivary Oxytocin and Cortisol Concentrations
Source: Front Hum Neurosci. 2017 Sep 14;11:430. doi: 10.3389/fnhum.2017.00430 (PMC5603757; doi:10.3389/fnhum.2017.00430)
Supplement: Supplementary file 1 [file Data_Sheet_1.docx]

Supplementary Material

**Choir versus solo singing: effects on mood and salivary oxytocin and cortisol concentrations.**

**Schladt T. M.^1&^, Nordmann G. C.^1&^, Kudielka B.M.^2^, de Jong T. R.^1^, Neumann, I. D^1^*.**

*** Correspondence:** Inga D. Neumann: inga.neumann@ur.de

# Supplementary Material

Singing task Cohort 1 (2014)

From G. F. Händel: **Messiah** (HWV 56):

Nr. 04. Chorus: And the glory, the glory of the Lord

No 19. Chorus: Behold the Lamb of God

No 39. Chorus: Hallelujah!

No 41. Chorus: Since by man came death

No 47. Chorus: Worthy is the Lamb

No 48. Chorus: Amen

Singing task Cohort 2 (2016)

From J. S. Bach: **Weihnachtsoratorium** (BWV 248):

Nr. 36. Chorus: Fallt mit Danken, fallt mit Loben

Nr. 42. Chorale: Jesus richte mein Beginnen

Nr. 43. Chorus: Ehre sei dir, Gott, gesungen

Nr. 46. Chorale: Dein Glanz all Finsternis verzehrt

Nr. 53. Choral: Zwar ist solche Herzensstube

Nr. 54. Chorus: Herr, wenn die stolzen Feinde schnauben
